# The Management of Posterior Malleolus Fractures in Unstable Ankle Injuries: Where Do We Stand Now?

**DOI:** 10.7759/cureus.32191

**Published:** 2022-12-05

**Authors:** Athanasios Serlis, Georgios Konstantopoulos, Panagiotis Poulios, Panagiotis Konstantinou, Konstantinos Ditsios, Michail Aftzoglou

**Affiliations:** 1 Orthopaedics and Trauma, Peterborough City Hospital, London, GBR; 2 Orthopaedics and Trauma, West Suffolk Hospital, London, GBR; 3 Orthopaedics and Trauma, Basildon University Hospital, London, GBR; 4 2nd Orthopaedic department, Aristotle University of Thessaloniki, Thessaloniki, GRC; 5 Upper Limb Surgery, Aristotle University of Thessaloniki, Thessaloniki, GRC; 6 Upper Limb Surgery, George Papanikolaou General Hospital of Thessaloniki, Thessaloniki, GRC; 7 Trauma, University Medical Center Hamburg-Eppendorf, Hamburg, DEU

**Keywords:** conservative, operative, surgery, trimalleolar fractures, ankle fractures, posterior malleolar

## Abstract

The evaluation and treatment of the posterior malleolus fracture in unstable ankle injuries remain a topic of controversy. The main objective of this systematic review was to examine the available literature and identify the variables that affect the management of posterior malleolar fractures and how these are related to the outcomes. To that end, a systematic review was performed based on the Preferred Reporting Items for Systematic Reviews and Meta-Analyses (PRISMA) guidelines. A comprehensive search of MEDLINE, Embase, and Cochrane Library was conducted. The search terms used were as follows: "posterior malleolar", "ankle fractures", "trimalleolar fractures", "ORIF", "surgery", "operative", and "conservative". The available studies were screened against the inclusion and exclusion criteria.

Based on the review of the available literature, we have concluded that the size of the posterior malleolar fragment is not an accurate indicator, and clinicians should consider other factors such as fracture configuration and articular surface congruity. Also, the risk for the development of post-traumatic arthritis increases when the joint surface is not restored regardless of the surgical intervention and fragment size.

The complications of posterior malleolus fractures necessitate evidence-based management. The assessment and the final treatment of these injuries in unstable ankle fractures should not be based on the traditional fragment-size parameters. Clinicians should assess the fracture configuration through imaging modalities and try to preserve the articular surface congruity so as to achieve optimal outcomes. Finally, more studies with high-level evidence are required in order to determine the most appropriate management pathway for these patients.

## Introduction and background

Ankle fractures constitute one of the most common lower extremity injuries, representing up to 5% of the presentations to emergency departments in the United Kingdom [1]. Trimalleolar ankle fractures account for 7% of the total lower limb injuries and are usually associated with a poor prognosis [2,3]. These injuries significantly impact the functional status of the patients and the healthcare system in general.

Posterior malleolar fragments result from rotational injuries as described by the Lauge-Hansen classification and are usually involved in complex and unstable ankle fractures [4]. Although the management of posterior malleolar fractures has been well described in the current literature, it remains controversial among orthopedic surgeons. Traditionally, the decision to fix posterior malleolar fractures was determined by the size of the articular fragment, ranging from 25% to one-third rule [5]. Presently, there are no clear indications regarding the operative and conservative treatment of these fractures, while it is commonly held that the fragment size should not be the only factor that will guide the clinician’s decision.

This systematic review aimed to evaluate how the management of posterior malleolar fractures (surgical vs. conservative) affects the outcomes of unstable ankle injuries. Also, this study assessed the elements that are crucial determinants of the decision-making process in the surgical fixation of posterior malleolar fractures, and treating surgeons should take them into consideration.

## Review

This systematic review was performed based on the Preferred Reporting Items for Systematic Reviews and Meta-Analyses (PRISMA) guidelines [6]. We conducted a comprehensive literature search on Embase, MEDLINE, and Cochrane Library. The following search terms were used to address the question of when to proceed to surgical intervention for posterior fragments in unstable ankle injuries: "posterior malleolar", "ankle fractures", "trimalleolar fractures", "ORIF", "surgery", "operative", and "conservative".

**Figure 1:**
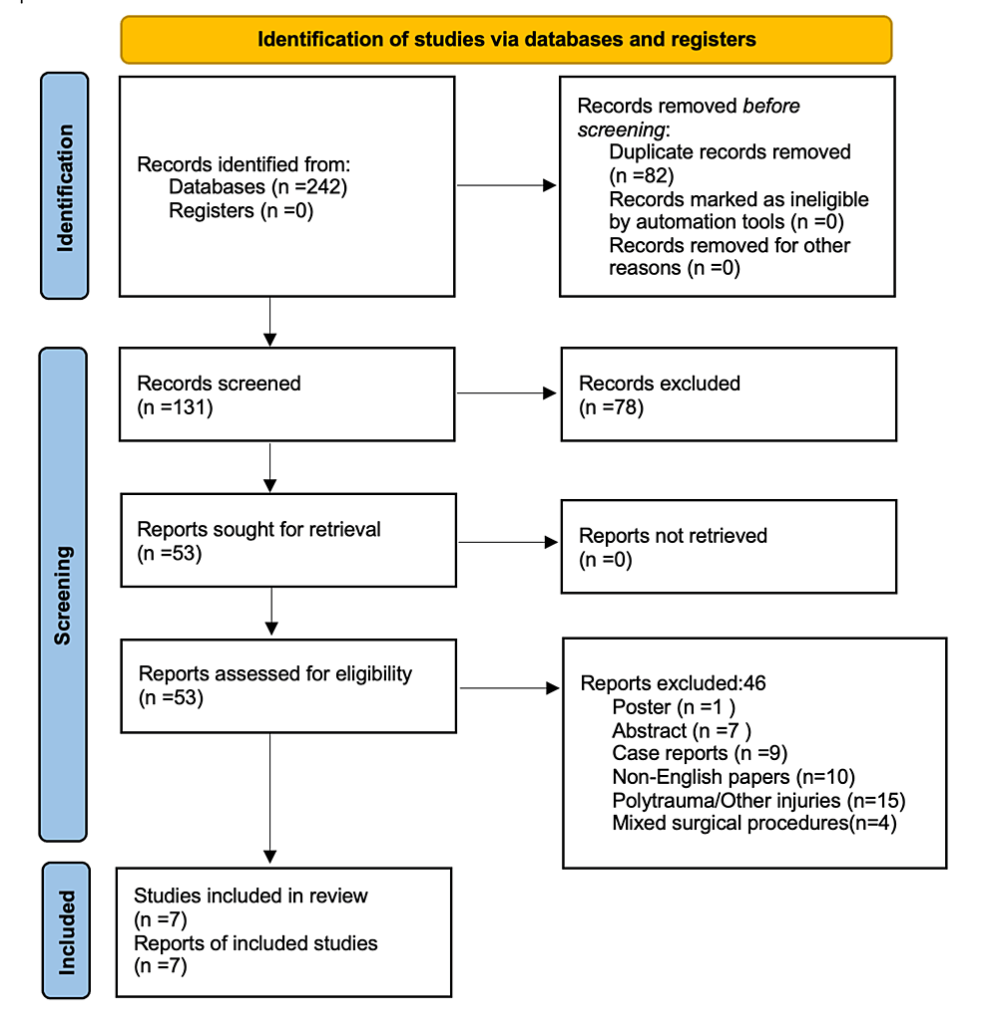
PRISMA flow diagram depicting the study selection process PRISMA: Preferred Reporting Items for Systematic Reviews and Meta-Analyses

Additionally, we evaluated all the available systematic reviews in the current literature to ensure that all the critical studies were included. The inclusion criteria of this systematic review were as follows: any study that included ankle fractures with the involvement of posterior malleolus fragment and their management, evaluation, and outcomes. Non-English papers, case reports, conference abstracts, and studies that included open and pathological fractures were excluded from this review. We also excluded studies involving posterior malleolus fractures associated with tibial shaft injuries and polytrauma patients (Figure 1). The available texts of the eligible studies were obtained. Finally, references from relevant studies were checked to identify other important studies in the literature. Studies older than 20 years were not considered eligible for this review, considering the recent changes in orthopedic practice and the technical advancements in managing these complex injuries.

Study characteristics, including the number of patients, age, gender, follow-up period, fixation method, and indications were recorded separately. Also, publication dates and authors, outcomes, functional scores, and study parameters were obtained from each study.

Blom et al. performed a retrospective study that included a total of 73 patients who had sustained an ankle fracture with involvement of the posterior malleolus in a four-year period [7]. The authors classified the patients into three groups according to the CT-based Haraguchi morphology. The follow-up period was two years, and the outcome was assessed based on FAOS (the Foot and Ankle Outcome Scores). The authors showed that Haraguchi type II ankle fractures (posterior malleolar fractures with medial extension) were associated with poorer functional outcomes compared to the other groups. They highlighted that evaluating the morphology of the posterior malleolus fragment is a crucial determinant of the outcome and should affect the decision-making process. The size of the posterior malleolus fragment as an indication for surgical fixation has been reviewed extensively in the literature; however, this study demonstrates the need for an appropriate assessment of the fracture morphology regardless of the fragment size.

Drijfhout van Hooff et al. conducted a retrospective study that reviewed the incidence of osteoarthritis and the long-term functional and radiological outcomes in 131 patients who had sustained ankle fractures with extension to the posterior malleolus [8]. They divided the patients into three groups according to the posterior malleolus fragment size (<5%, 5%-25%, and >25%) and the presence of postoperative step-off. Their findings showed that osteoarthritis occurred more frequently in larger fragment sizes (>5%) and cases with step-off >1 mm in the tibiotalar joint surface. However, no differences were reported in the incidence of osteoarthritis when the posterior malleolus fracture was fixed. Also, the functional outcome and range of motion were similar in all three groups. Despite the retrospective nature of this study and the poor compliance of patients with the follow-up, which increases the risk of bias, the researchers showed that the anatomical restoration of the joint surface is associated with less risk of osteoarthritis regardless of the fragment size and the fixation of the posterior malleolus fracture.

De Vries et al. included 45 patients who had sustained ankle fractures with posterior malleolar involvement in their study [9]. The authors sought to identify the long-term results and assess the necessity of surgical fixation for posterior malleolus fragments smaller than 25% in ankle fractures. They used an ankle fracture scoring system and a numeric pain scale to identify the differences between the outcomes in different groups. Results showed no significant difference between surgical and conservative management regarding long-term patient outcomes. More specifically, in 38% of the patients, the large posterior malleolar fragment (>25%) was treated conservatively, and this group showed similar outcomes compared with the group where the posterior malleolar was fixed. However, no statistical analysis was performed on the latter group due to the small sample size. The authors reported that functional outcomes were worse when posterior malleolus fractures were associated with ankle fracture-dislocation after 13 years of follow-up.

Mingo-Robinet et al. conducted a retrospective study that included 45 patients treated with surgical fixation of a trimalleolar ankle fracture over a period of four years [10]. The main goal of this study was to correlate the outcome with the size of the posterior malleolar fragment. Better results were obtained in patients with fragment size <25% based on the American Orthopedic Foot and Ankle Society (AOFAS) score; however, this study showed that anatomical reduction of the posterior malleolar fractures did not lead to better functional outcomes compared to the nonanatomic reduction group. Despite the absence of statistically significant results, the authors highlighted that small fragments (<25%) are associated with less favorable outcomes when anatomical reduction is not achieved. This finding is supported by Langenhuijsen et al., who showed that restoration of the joint congruity in posterior malleolar fragments >10% improves the long-term outcomes, with or without surgical fixation [11].

Xu et al. retrospectively reviewed the treatment outcomes in ankle fractures with posterior malleolar involvement [12]. The authors included 102 patients treated at five trauma centers in China over a period of nine years; they recorded patient satisfaction rates based on AOFAS scores and employed imaging scales to evaluate the degree of arthritis. One of the main findings of this study was that conservative management of posterior malleolar fragments with a size of less than 25% has similar outcomes with surgical repair. Additionally, the authors showed that the choice of surgical technique (front-to-back, back-to-front) has no impact on the outcome. They also highlighted that the treatment choice (surgical vs. conservative) of the posterior malleolar fragments with a size of less than 25% does not affect the arthritis score and patients’ satisfaction rates. Finally, the restoration of the articular surface was associated with a better prognosis in all three groups (<10%, 10%-25%, and >25%); the authors concluded that anatomical reduction of the posterior malleolus is crucial regardless of the treatment choice.

Tejwani et al. conducted a retrospective study involving 456 patients who had sustained unstable ankle fractures over a period of five years [13]. In 54 patients, there was fracture extension to the posterior malleolus, and fixation was performed in one-third of them. The main strengths of this study were as follows: a similar rehabilitation protocol was followed for all patients, and the AOFAS score was utilized to assess their functional outcomes. Results showed statistically significant differences in the long-term outcomes of ankle fractures between the two groups (with and without posterior malleolar involvement). More specifically, functional outcomes were worse in the posterior malleolus group at one year, while results were similar during the two-year follow-up period. The authors proposed that posterior malleolus fractures might be related to high-energy injuries; thus, differences in outcomes at one-year follow-up could be justified.

Bua et al. analyzed the outcomes in 320 patients who were treated surgically for ankle fractures with posterior malleolar involvement [14]. This study was conducted at a major trauma center; the large sample group and the two-year follow-up period confer reliability to its results. The statistically significant results support that fixation of the posterior malleolus provides better patient outcomes; however, the complication rates were almost 11% higher in the fixation group. One of the most important findings of this study was that reoperation rates were 10% higher in the fixation group compared with the unfixed group. The authors highlighted that this resulted from metalwork-related complications. Additionally, this study showed that posterior malleolus fixation provides better results than syndesmotic fixation; this finding is also supported by Gardner et al., who showed that posterior malleolus fixation provides better results regarding syndesmotic stiffness compared to the syndesmotic screw [15]. Furthermore, Gardner et al. demonstrated that foot and ankle surgeons consider other factors, such as subluxation and comminution, to be more critical than the fragment size. De Vries et al. also showed similar outcomes between the surgical and conservative treatment for large posterior malleolar fragments (<25%). Despite the lack of sufficient statistical analysis, the findings of this study propose that the cut-off size of 25% should not be used as an indicator for the fixation of the posterior malleolus fractures (Table 1).

**Table 1:** Studies comparing management options and outcomes of posterior malleolus fractures

Paper	Number of patients included in the study	Number of posterior malleolar fractures treated surgically	Mean follow-up period	Level of evidence	Outcomes
Blom et al. [7]	73	70	24 months	III	Improved clinical scores
Drijfhout van Hooff et al. [8]	131	N/A	6.9 years	IV	Improved clinical scores
De Vries et al. [9]	45	28	13 years	III	Improved clinical scores
Mingo-Robinet et al. [10]	45	45	24 months	II	Improved clinical scores
Xu et al. [12]	102	42	33.8 months	III	Improved clinical scores
Tejwani et al. [13]	456	20	24 months	III	Improved clinical scores
Bua et al. [14]	320	160	24 months	III	Improved outcomes but increased reoperation and hardware risks

On the contrary, McDaniel and Wilson suggested that fragment sizes >25% should be treated as they are associated with better results [16]. However, this study had a small sample size, and there is evidence of selection bias. Finally, recent studies have shown that imaging modalities are essential for the final decision-making to manage posterior malleolus fractures. More specifically, Gonzalez et al. highlighted the importance of the externally rotated lateral view in complex ankle injuries to appropriately evaluate the degree of displacement of the posterior malleolar injuries [17].

Limitations

The limited available data in the current literature regarding the indications of surgical fixation of posterior malleolus fractures increase the risk of bias significantly. Also, there is evidence of selection bias due to the variability of the patient population. Additionally, different surgical techniques have been used by each group of surgeons in the included studies, which can affect the outcomes. Moreover, there is a risk of bias due to the variability of the functional outcome scores and the follow-up periods implemented in each study. The retrospective nature of the included studies also increases the risk of selection bias; thus, the surgical indications and outcomes might not correlate with the acute clinical setting. Additionally, three of the included studies did not have adequate follow-up periods, and each used different functional outcome scores and recovery programs. Also, we only included studies in the English language, and some studies with a high level of evidence might have been missed out.

## Conclusions

Based on the documented outcomes related to posterior malleolus fractures in the literature, there is considerable debate about the most appropriate management pathway for these patients. This study demonstrates that the fragment size should be considered a secondary indicator as it has been traditionally used. The current evidence suggests that orthopedic surgeons should assess the fragment size in correlation with the fracture configuration and articular surface congruity. Also, our findings reveal that the incidence of osteoarthritis, the major long-term complication of these injuries, increases when the articular surface congruence is not preserved, regardless of the fragment size and surgical intervention.

Moreover, there is a direct correlation between radiographic osteoarthritic changes and fracture complexity, at a ratio of 1:3. Interestingly, it still needs to be clarified as to when radiographic osteoarthritis becomes symptomatic and necessary to be treated. Undeniably, more high-quality studies with larger sample sizes are required to provide valuable data to help manage these complex but common injuries in everyday practice.
